# Nudges for Judges: An Experiment on the Effect of Making Sentencing Costs Explicit

**DOI:** 10.3389/fpsyg.2022.889933

**Published:** 2022-05-20

**Authors:** Eyal Aharoni, Heather M. Kleider-Offutt, Sarah F. Brosnan, Morris B. Hoffman

**Affiliations:** ^1^Department of Psychology, Georgia State University, Atlanta, GA, United States; ^2^Department of Philosophy, Georgia State University, Atlanta, GA, United States; ^3^Neuroscience Institute, Georgia State University, Atlanta, GA, United States; ^4^Center for Behavioral Neuroscience, Atlanta, GA, United States; ^5^Retired, Second Judicial District, Denver, CO, United States

**Keywords:** punishment, sentencing, cost–benefit, judge, decision making

## Abstract

Judges are typically tasked to consider sentencing benefits but not costs. Previous research finds that both laypeople and prosecutors discount the costs of incarceration when forming sentencing attitudes, raising important questions about whether professional judges show the same bias during sentencing. To test this, we used a vignette-based experiment in which Minnesota state judges (*N* = 87) reviewed a case summary about an aggravated robbery and imposed a hypothetical sentence. Using random assignment, half the participants received additional information about plausible negative consequences of incarceration. As predicted, our results revealed a mitigating effect of cost exposure on prison sentence term lengths. Critically, these findings support the conclusion that policies that increase transparency in sentencing costs could reduce sentence lengths, which has important economic and social ramifications.

## Introduction

Criminal court judges are explicitly trained to consider the expected benefits of their sentences, such as retribution, incapacitation, and deterrence (American Law Institute, 2017), but not the costs (Flanders, 2012a). This may be by design, as costs are commonly viewed as extraneous to the sentencing process and are offloaded to other levels of government (State of Connecticut v. Bell, 2011; United States v. Park, 2014). But insulating judges from sentencing costs does not make these costs go away. For example, the direct monetary cost of incarcerating a single inmate averages $33,000 per year (Mai and Subramanian, 2017), a figure that rivals college tuition. This says nothing of the many collateral consequences of incarceration for offenders and their families, such as loss of income (e.g., Kirk and Wakefield, 2018), or the possible criminogenic consequences of incarceration (e.g., Stemen, 2017), which disproportionately affect disadvantaged communities (Bierschbach and Bibas, 2017).

When judges are exposed to the benefits of a sentence but not the costs, they might punish more than when costs and benefits are considered in concert. Facing the unanticipated consequences of high incarceration rates, this prediction has fueled recent policy efforts (e.g., California Assembly Bill 1474) to increase transparency in sentencing by disclosing the cost of incarceration to judges at the time of sentencing (Ewing, 2018; Alpert, 2021). Similar policies have already been adopted in a few other jurisdictions, such as Colorado and Missouri (Flanders, 2012b; Colorado Revised Statutes, 2021). But what effect, if any, will such exposure have on judicial sentencing decisions? We consider three rivalrous theoretical predictions: deontological, economic, and cognitive. Deontological, or duty-based, theories assert that punishment judgments should be determined exclusively by the principle of just deserts (i.e., what the offender deserves with respect to the wrongfulness of his transgression; Hart, 1968; American Law Institute, 2017) In this view, the material consequences of the punishment are irrelevant, so their utility should be zero. Economic theories, in contrast, assume that decision costs and benefits are potentially relevant and so sentencing cost information could have a mitigating effect on sentences, but only if it contains added value to the decision-maker (Becker, 1968).

So, if judges are already aware of sentencing costs, or simply do not value them, then exposure to cost information should not affect their punishment judgments. Conversely, if cost exposure does reduce their punishments, this would imply that they value cost information but their consideration of that information hinges on their access to it. This latter view is consistent with cognitive theories that preferences are sensitive to contextual and psychological factors, such as availability of information (Tversky and Kahneman, 1973). According to this perspective, if judicial valuation of sentencing costs is conditional upon their contextual salience (i.e., reminding of costs), then exposure to relevant cost information, such as the negative consequences of incarceration, should reduce their sentences relative to the status quo. Confirmation of this hypothesis would have implications for incarceration rates and, therefore, would inform policy debates about what types of information should and should not be available to judges during sentencing.

Systematic tests of this hypothesis, however, are sorely lacking. Most studies on the effect of sentencing cost exposure have examined attitudes among laypeople. These studies demonstrate that exposure to information about sentencing costs reduces the severity of punishment recommendations or support for punitive policies (Thomson and Ragona, 1987; Gottlieb, 2017; Aharoni et al., 2018, 2019, 2020). Judges and prosecutors might be less sensitive to sentencing cost information than laypeople because they know more about those costs, or because they consider those costs to be irrelevant. However, only a few studies have examined the effects of punishment cost exposure on judges and prosecutors. In one recent vignette experiment conducted with a national sample of prosecutors (Aharoni et al., 2021), we found that when prosecutors were insulated from sentencing cost information, their prison sentence recommendations for an offender convicted of drug trafficking were almost a third longer than sentences rendered following exposure to brief information about the cost of incarceration (Aharoni et al., 2018, 2020). Exposure to a fiscally equivalent benefit of incarceration had no impact on prosecutors. We concluded that prosecutors implicitly value incorporating sentencing costs but selectively neglect them unless they are made explicit, and this tendency could have a consistently aggravating effect on the sentencing recommendations they make to judges (Aharoni et al., 2021).

Only one study has examined cost framing in professional judges. In that study, judges from a variety of jurisdictions were exposed to true information about the direct cost of incarceration for a rape case and rendered sentences that were about 30% shorter than those exposed to no- or low-cost information (Rachlinski et al., 2013). This important finding raises several new questions: Would the cost salience effect occur in response to a wider variety of negative consequences of incarceration that might be relevant to judges in addition to purely monetary ones? Would it generalize to other crime types? And would it survive the use of real-world sentencing guidelines, which impose statutory constraints on the presumptive and allowable sentencing range in many jurisdictions?

If cost salience mitigates judicial sentences, this evidence would be consequential for policy efforts aiming to disclose (or block) cost information in court (e.g., CA bill 1474). One concern about such policies is that they could result in arbitrary disparities in sentencing because different judges might interpret and value the costs differently (Flanders, 2012b). This question can be directly tested by evaluating potential differences in variance between judges who are exposed to cost information versus those not exposed.

This article reports a test of these questions in a sample of state judges in Minnesota. Minnesota is one of 25 U.S. states that employs sentencing guidelines, which are designed to increase standardization between judges and their prison sentences. If prison sentencing cost information exhibits measurable effects within the constraints of a guideline framework, its influence in states without guidelines is likely to be at least as strong.

Using an experimental vignette method, we presented a case of aggravated robbery to our judge participants, and using random assignment, we manipulated the presence or absence of various negative consequences of incarceration, including the direct monetary cost of incarceration but also the negative impact on the defendant’s family, employability, and probability of reoffending. Participants responded using a real prison sentencing range derived from Minnesota statutory law. We predicted that exposure to information about the plausible negative consequences of incarceration would reduce prison sentencing judgments among judges relative to a (status quo) condition with no cost information, suggesting that judges ultimately value cost considerations but neglect to consider them under the status quo. Evidence for this hypothesis would represent an important step in identifying the hidden drivers of high incarceration rates and how best to manage them.

## Materials and Methods

### Participants

Participants were 87 Minnesota state judges with at least 6 months of experience on the bench. Sixty-two were recruited from virtual workshops in the Minnesota Annual Conference of State Judges in December 2021. All MN state judges are invited to the conference and most (~2/3) participate. The remainder were recruited by workshop participants who forwarded the survey invitation to colleagues on their judicial district mailing lists in February 2022. This strategy did not lend itself to assessment of response rates since we could not obtain complete records of how many participants received a survey invitation. What is known is that all those who responded to the electronic consent question completed the survey.

Sample composition was 50.58% male, 44.83% female, similar to MN base rates^1^; with a mean age of 55.64 years (SD = 8.70) and *M* = 9.94 years of judicial experience (SD = 7.38). These attributes did not differ statistically between conditions (See Table 1). 80.46% reported working across units, and the remainder worked in a specialized unit (e.g., felony, misdemeanor, juvenile justice, and family law). Less than 4% reported a caseload that did not include criminal trials (e.g., civil or appellate judges). We did not exclude these judges because civil and appellate judges commonly have some experience with criminal law. The overall sample leaned slightly liberal at *M* = −0.96 (SD = 1.04), though this may be on par with MN base rates (Bonica and Woodruff, 2012). Ethnicity and race were not collected. Since conference and survey participation were voluntary, it is possible that certain demographic traits were disproportionately represented, introducing possible selection bias, but we do not have data to address this possibility.

**Table 1:** Relations of demographic variables to independent variable (cost information) and dependent variable (sentence length).

	Cost information	Sentence length	Cost present		Cost absent		Difference statistic (DS)
	*r* (*p*-value)	*r* (*p*-value)	*M* (*SD*)^a^	N	*M* (*SD*)^a^	N	DS^b^ (*p*-value)
Gender (*f* = 0; *m* = 1)	0.18 (0.10)	0.01 (0.92)	55.81%	43	37.50%	40	2.79 (0.10)
Age (years)	0.07 (0.57)	0.07 (0.54)	55.08 (8.08)	38	56.30 (9.41)	35	0.58 (0.57)
Experience (years)	0.03 (0.81)	0.03 (0.80)	9.74 (7.68)	43	10.15 (7.14)	41	0.25 (0.81)
Political ideology	−0.04 (0.76)	0.00 (0.98)	−0.93 (1.05)	42	−1.00 (1.04)	40	−0.31 (0.76)

All values of *p* ≥ 0.10.

a Denotes mean (M) and standard deviation (SD) except for Gender, which shows percentage of the sample that is female.

b All difference statistics are represented by (independent samples) *t*-values except for Gender, which shows a chi-squared statistic.

### Design and Hypotheses

This study used a two-group design. Participants were randomly assigned to one of two cost conditions: information about negative consequences of incarceration was either present or absent. Based on previous research, our hypothesis was that sentencing judgments would be lower when this information was present than absent.

### Materials and Procedures

Prior to their workshop presentation, participants were invited to participate in an anonymous 5-min web survey on “legal decision-making.” Using the Qualtrics survey platform, we presented a fictitious case summary describing an aggravated robbery. First, participants were instructed:

Imagine you are presiding over a case of Aggravated Robbery, a level 8 felony. You will read a case summary about an adult defendant who has been found guilty, then you will decide on his sentence. Then you will be asked questions about yourself and about the case, so please read attentively.

The case summary was constructed to assure participants that the defendant was factually guilty, and the vast majority (95.4%) later agreed with a forced-choice statement that there was enough evidence to support the defendant’s conviction (disagree vs. agree). The use of a dangerous weapon and a prior offense were stipulated to ensure that the presumptive sentence, according to MN statute, would be prison. The case summary stated:

Joseph, a 35 years-old man, was charged and convicted after trial of one count of aggravated robbery in the first degree. He accosted a 39 years-old female patron behind a gas station, demanding her wallet. When she hesitated, Joseph swung a crowbar at her face, narrowly missing her jaw, then attempted to flee with her wallet containing $300. A security guard apprehended Joseph on the scene until police could make an arrest. The incident was captured on security footage, and Joseph confessed. Ten years ago, Joseph completed a sentence for a prior assault with a knife. He has a handful of other misdemeanor convictions, none violent, and no other prior felony convictions.

Participants in the treatment condition received an additional statement about the negative consequences of incarceration. The statement was intended to cover an array of plausible consequences, and participants in this condition confirmed their plausibility on a 5-point ordinal scale from “not at all plausible” to “very plausible”, *M* = 3.00 (*SD* = 0.90). The manipulation stated:

*Incarcerating Joseph would likely have the following negative consequences:*

- *increase the financial burden on taxpayers for each year that he is incarcerated*
- *place an emotional and financial burden on Joseph’s family*
- *reduce Joseph’s employability after he is released*
- *increase Joseph’s odds of committing other serious crimes in the future*

Our goal was to test the salience of judge’s general knowledge about a variety of negative consequences of incarceration rather than specific factual details. Therefore, we sacrificed some details that might appear in actual arguments made in a sentencing hearing, such as information that might clarify the defendant’s current level of dangerousness. This decision, though limiting the study’s ecological realism, assured that confirmation of our hypothesis could not be explained as merely the result of particular anomalous, confounding, or contestable details.

Next, the dependent measure was delivered, asking participants to indicate how much prison time should be imposed on a slider scale that ranged from “42 mo. or less” to “96 mo. or more.” The instructions specified the true presumptive guideline range, based on realistic assumptions about the defendant’s criminal history score and the severity level of his index crime (Minnesota Sentencing Commission, 2021).

Assume Joseph's criminal history score is 2, making the presumptive guideline sentence range 58–81 months in prison. Further assume you decided to send Joseph to prison. Based solely on these facts, how many months in prison will you impose for this offense? Drag the slider anywhere on the scale.

We made the scale wider than the guideline range because under Minnesota law, judges may depart from the guideline range.

Next, credibility checks were administered to assess plausibility of the evidence and the consequences of incarceration. Then we assessed participants’ explicit attitudes about whether “judges should consider the negative consequences of the sentence before deciding how much an offender should be sentenced,” using a 7-point scale from “strongly disagree” (−3) to “strongly agree” (−3). We assessed self-reported political ideology using a 7-point scale from “very liberal” (−3) to “very conservative” (+3). Finally, we collected information about age, gender, specialization, and years of judicial experience. Median survey completion time was 4.02 min. All study procedures were approved by the university’s ethical review board and conditioned on informed consent. All data were analyzed using IBM SPSS v. 26.

## Results

**Figure 1:**
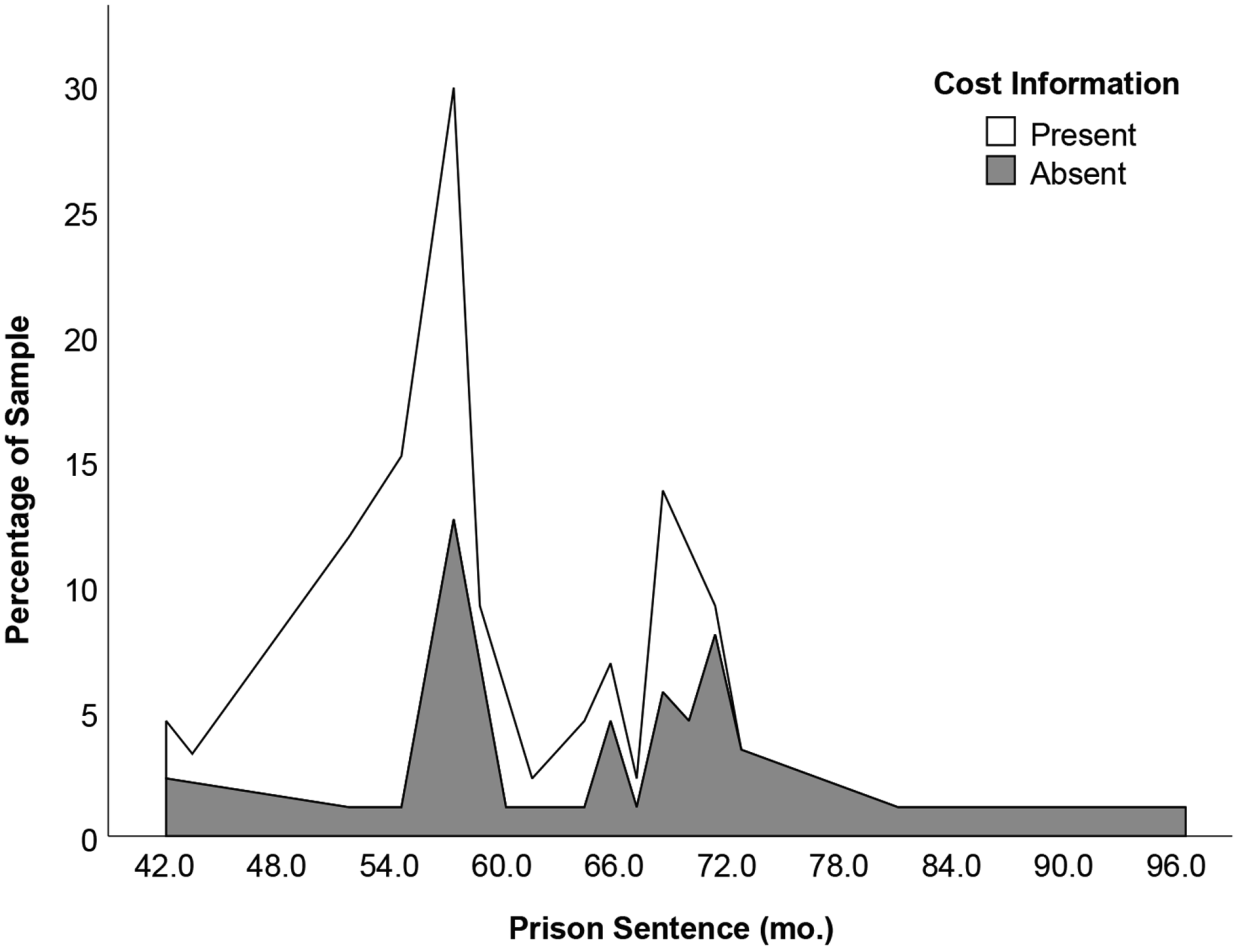
Histogram of prison sentences colored by cost information (present vs. absent), interpolated across 43 bins. Plot shows a disproportionate representation of sentences from the present condition on the lower end of the sentencing scale. Y-axis represents the percentage of total number of responses.

Did exposure to plausible negative consequences of incarceration precipitate a sentencing reduction? Using a one-way ANOVA, a main effect of cost salience emerged, *F*(1, 85) = 4.14, *p* = 0.045, $\eta_{p}^{2}$ = 0.05. Consistent with our hypothesis, judges exposed to the list of plausible negative consequences of incarceration imposed prison sentences that were significantly shorter (*M* = 61.56 months, SE = 1.28, 95% CI [59.02, 64.09]) than those not exposed (*M* = 65.22, SE = 1.26, 95% CI [62.70, 67.72]). This difference amounts to 15.87% change within the presumptive sentencing range of 58–81 months. According to Levene’s test of equality of variances, we also assessed whether the variation between judges’ sentences was influenced by exposure to the costs. We did not detect any difference between these variances, *F*(1, 85) = 1.98, *p* = 0.164 (see Figure 1).

A few judges (7) made a downward departure below the presumptive range (and 1 departed above that range), but more of these individuals (4 of the 7) were in the cost absent condition. Indeed, when we exclude those who departed from the presumptive range, the size of the main effect of cost exposure shows a modest increase, *F*(1, 76) = 5.76, *p* = 0.019, $\eta_{p}^{2}$ = 0.07, *M* = 62.97, SE = 0.89, 95% CI[61.20, 64.75], relative to no exposure, *M* = 66.02, SE = 0.92, 95% CI[64.22, 67.86]. This pattern supports the inference that our cost manipulation shifted their judgments *within* the presumptive range. Once again, error variances did not differ between conditions, *F*(1, 76) = 0.974, *p* = 0.33.

When asked about their explicit attitudes on whether judges should consider the negative consequences of incarceration in their sentencing decisions, participants agreed that they should, *t*(86) = 5.70, *p* < 0.001, *M* = 0.91 (SD = 1.48). However, this attitude was not affected by exposure to our cost manipulation, *t*(43) = 1.02, *p* = 0.31, suggesting a level of stability that pre-existed study participation.

Last, our independent and dependent variables were not associated with any of our demographic variables, precluding evidence of moderation or mediation (see Table 1).

## Discussion

The purpose of this study was to test the impact of cost information on prison sentencing judgments made by professional judges. As predicted, judges leveled harsher prison sentences in the absence of information about plausible negative consequences of incarceration. By inference, these judges discounted the negative consequences of imprisonment unless they were made salient. The effect size was modest, but an effect of any size is remarkable given the large number of other factors that undoubtedly explain variance in prison sentencing.

According to the deontological (duty-based) punishment theory, judges should discount or outright ignore information about the negative consequences of incarceration when forming sentencing judgments (see Hart, 1968). Indeed, when left to their own devices—that is, when not prompted by salient cost information—our findings suggest that they do just that. Economic theories, in contrast, predict that punishment decisions should be responsive to their costs—even without prompting—but only if judges are aware of and value those costs (see Becker, 1968). In our study, the negative consequences presented were of the ordinary sort with which any state judge would already be readily familiar. Even if judges were inspired to change their underlying sentencing preferences upon exposure to the brief cost information presented in our study (see Greenberg and Spiller, 2016), this should have been reflected in their explicit attitudes about sentencing, but participants across both conditions equally and positively endorsed the notion that sentencing judges should consider the potential negative consequences of incarceration. So the fact that exposure to brief information about those consequences was sufficient to exert any mitigating influence on their sentencing judgments suggests that while they implicitly value the decision costs, they neglect to consider them unless they are made salient. In short, their consideration of prison sentencing cost information is conditional upon their access to it.

This pattern of results comports well with cognitive perspectives. For instance, scholarship on the heuristics and biases framework suggests that punishments will be sensitive, not just to the overt utility of the cost/benefit information but also extra-legal, contextual factors like its availability (Tversky and Kahneman, 1973; Bennett, 2014). In this view, decision-makers follow an “out of sight, out of mind” rule whereby the default decision-making mode is to only consider information that is made explicit at the time of decision (Aharoni et al., 2020, 2021). This tendency to neglect decision costs seems especially likely in criminal punishment decisions, wherein judges are often tasked to evaluate immoral acts that violate their sacred values. Sacred values are highly resistant to compromise because their utility is ostensibly infinite (Tetlock, 2003). Yet, when prompted to consider the negative consequences of sacred value claims, research has shown that some degree of moral compromise may occur (Baron and Leshner, 2000).

Our results complement existing research on punishment cost discounting using a distinctive sample of state judges. Our findings are consistent with other studies on sentencing cost discounting in judges (Rachlinski et al., 2013), prosecutors (Aharoni et al., 2021), and laypeople (Thomson and Ragona, 1987; Gottlieb, 2017). Our study extends this research in three key ways. It uniquely shows that the cost salience effect (1) can occur in response to a wider variety of negative consequences of incarceration that might be relevant to judges than has previously been demonstrated, (2) can occur in response to other serious crime types, namely, aggravated robbery, and (3) can survive the use of real-world sentencing guidelines, which impose constraints on the presumptive and allowable sentencing range.

Considering real-world relevance, our data speak to policies, such as CA AB 1474, that would require the disclosure of sentencing cost information to judges at the time of sentencing (Alpert, 2021). This information could be included in the judge’s presentencing report alongside the expected benefits of the sentence. Scholars have expressed concern that such policies would create disparities in sentencing between judges (Flanders, 2012a). However, our data do not support this inference since the variation in participants’ sentences (Levene’s test) did not statistically differ between conditions. To the contrary, providing cost and benefit information to judges carries the potential to foster more consistency in judgments since such information would no longer be left to the judge’s imagination. Such a strategy would not seem to violate established doctrine on the purposes of punishment. Indeed, the current edition of the Model Penal Code’s section § 1.02(2b) on the purposes of punishment includes specific provisions to increase transparency in sentencing and to ensure adequate resources are available for sentences (American Law Institute, 2017). Sentencing cost information would also seem to be justifiably relevant to arguments made by the defense. Defense lawyers, therefore, could benefit from training about presenting sentencing cost information to judges, provided that evidence meets the criminal procedural rules of that jurisdiction.

Our study conclusions are necessarily limited by our methodological choices. First, our sample is not necessarily generalizable to judges in other jurisdictions. Minnesota’s particular sentencing rules and ranges will almost certainly differ in some respects from those in other states. We restricted our sample to Minnesota partly because it is a guideline state, permitting a test of the claim that guideline ranges will neutralize any effects of sentencing cost information. Restricting our sample to a single jurisdiction also increases our ability to generalize to sentencing behavior more broadly within that jurisdiction. Yet, despite the additional constraints built into our methodology, the sentencing behavior observed in our experiment replicates that of legal practitioners in more geographically diverse samples (Rachlinski et al., 2013; Aharoni et al., 2021).

In addition, our study was limited to a single crime type. We would not necessarily expect cost framing effects to be as strong among the most serious crimes, such as capital offenses, but future research could test this hypothesis empirically. Meanwhile, the fact that these effects have now been observed in a case of aggravated robbery, and elsewhere in a case of rape (Rachlinski et al., 2013) suggests that they are not limited to the least serious crimes.

Our expected sample size was modest and prevented us from testing the impact of making benefits salient. That being said, our previous research has shown that the sentencing recommendations of prosecutors, who often face professional and public incentives to negotiate for tough penalties, are insensitive to exposure to information about the benefits of incarceration (Aharoni et al., 2021). The same pattern has been found among lay judges (Aharoni et al., 2020). It may simply be that these benefits, unlike costs, are already saliently built into the theories undergirding criminal punishment. If prosecutors and laypeople are insensitive to benefits information, we might expect professional judges to be too.

Future research should consider which types of negative consequences of their sentences matter most for judges, such as financial costs to taxpayers versus collateral consequences to the offender’s family. Importantly, answers to these questions could depend on how these consequences are framed and measured. Previous research suggests that market pricing frames reduce support for social initiatives, at least when using self-report measures (Tetlock et al., 2000). Our own research on self-reported punishment attitudes in prosecutors (Aharoni et al., 2021) and laypeople (Aharoni et al., 2018) confirm this (i.e., participants did not express support for consideration of sentencing costs), but our implicit measures of their punishment judgments revealed a sensitivity to cost exposure nonetheless. Any thorough characterization of the decision factors that judges actually value must account for these differences in framing and measurement.

We kept the information about the defendant and the negative consequences of incarceration quite brief to guard against intrusion of potentially confounding details. However, this decision necessarily limits the ecological realism of our stimuli with respect to actual sentencing hearings. Future research should thus consider richer, more naturalistic descriptions, such as the defendant’s employment history and dangerousness level.

Limitations aside, our study findings support the prediction that, without access to explicit cost cues, professional judges are more punitive than they would be under more informationally transparent conditions. Importantly, the question at hand is not whether scientists can get judges to be more lenient. It is whether judicial sentencing judgments could be systematically biased by their choice architectures, and what, if anything, can shift those biases. Our study found that exposure to brief and plausible cost information may indeed shift those biases. This study, thus, contributes to emerging policy debates on transparency in sentencing.

## Data Availability Statement

The datasets presented in this study can be found in online repositories. The names of the repository/repositories and accession number(s) can be found at: https://osf.io/h7py6/, Open Science Framework.

## Ethics Statement

The studies involving human participants were reviewed and approved by Institutional Review Board, Georgia State University. The patients/participants provided their written informed consent to participate in this study.

## Author Contributions

EA, HK-O, SB, and MH conceived the project and developed the empirical approach. EA performed the data collection and analysis and wrote the first draft of the manuscript. All authors contributed to discussions about the paper’s focus, proposed edits, and approved the final version of the manuscript for submission.

## Conflict of Interest

The authors declare that the research was conducted in the absence of any commercial or financial relationships that could be construed as a potential conflict of interest.

## Publisher’s Note

All claims expressed in this article are solely those of the authors and do not necessarily represent those of their affiliated organizations, or those of the publisher, the editors and the reviewers. Any product that may be evaluated in this article, or claim that may be made by its manufacturer, is not guaranteed or endorsed by the publisher.

## Footnotes

^1^ https://https://www.mncourts.gov/
